# Biochar—A Filler in “Bioplastics” for Horticultural Applications

**DOI:** 10.3390/ma17246208

**Published:** 2024-12-19

**Authors:** Krystyna Malińska, Danuta Dróżdż, Przemysław Postawa, Tomasz Stachowiak

**Affiliations:** 1 Faculty of Infrastructure and Environment, Czestochowa University of Technology, ul. Dabrowskiego 73, 42-201 Czestochowa, Poland; danuta.drozdz@pcz.pl; 2 Faculty of Mechanical Engineering, Czestochowa University of Technology, Al. Armii Krajowej 21, 42-201 Czestochowa, Poland; przemyslaw.postawa@pcz.pl (P.P.); tomasz.stachowiak@pcz.pl (T.S.)

**Keywords:** biochar, filler, bioplastics, horticulture, mulch films, plant accessories

## Abstract

Biochar is attracting a lot of attention as it is considered a novel, renewable, and bio-based filler that can be used specifically for developing and manufacturing “bioplastics” for growing plants such as mulch films and plant accessories. The manufacturing of “bioplastics” uses biopolymers but also various additives such as fillers, which are primarily used to replace some of the expensive biopolymers in a biocomposite composition and/or to improve the mechanical properties of the final products. This review aims to demonstrate the applications of biochar as a filler in bioplastics, specifically for horticultural uses; summarize the most recent findings; and discuss future research directions. With this review, we address some of the most important issues related to the requirements for biochar as a filler for bio-based and biodegradable plastics, the effect of biochar properties and loading rates on the properties of biocomposites, and the suitability of biochar for manufacturing of “bioplastics” for horticultural use. We also discuss the advantages as well as challenges and limitations to the use of biochar for manufacturing bio-based and biodegradable plastics for horticultural uses.

## 1. Introduction

### 1.1. Biochar

Biochar is a solid, charcoal-like, carbonaceous material obtained from pyrolysis of plant or animal-derived biomass feedstock. Due to its wide variety of properties, biochar demonstrates a number of applications in many areas, particularly in agriculture, to improve soil properties and increase crop yields [1,2,3,4,5]. In 2023, almost 77% of the produced biochar was used in agriculture, with the rest being used in animal husbandry and industry [6]. One of the most popular applications of biochar is horticulture, where biochar is mostly used as a soil enhancer [7,8,9] to improve soil physical (soil structure, bulk density, soil porosity, and infiltration, aggregate stability), chemical (soil CEC, pH, water holding capacity, nutrient storage, and availability), and biological (soil organic matter production, microbial activity, mycorrhizal colonization) properties [10]. Biochar is also considered a potential replacement for peat in growing media for ornamentals and vegetables [11,12,13]. What is more, biochar can be a growth substrate or co-substrate in hydroponic systems where leafy vegetables (e.g., lettuce) [14,15] or fruits (e.g., strawberry) are grown [16]. With all these applications and a continuously increasing interest in biochar, it is anticipated that the biochar market will grow by 14% between 2024 and 2030 [17].

But beyond the direct in-soil applications, there is also a great potential for biochar as a sustainable and renewable filler [18,19,20] to be incorporated into composites or biocomposites to develop plastics or bioplastics to be used in agriculture and horticulture, specifically mulch films, seedling pots, and trays or plant accessories (e.g., supports, ties, clips, strings, threads, nets) [20,21,22,23,24]. All of these plastics/bioplastics are commonly used in crop production to grow edible plants in open fields as well as in greenhouses or tunnels. What is more, according to the most recent studies, biochar can be used as a filler for biodegradable mulch films and a fertilizer carrier at the same time, e.g., a fertilizer carrier of urea [25]. This opens more options for bio-based and biodegradable mulch films, which are now included as soil enhancers in the Fertilising Product Regulation 2019/1009 [26,27,28].

### 1.2. Plastics and Bioplastics for Crop Production

Generally, “plastics” are referred to as conventional fossil-derived materials that do not undergo biodegradation, whereas “bioplastics” are defined as bio-based (fully or partially) or biodegradable or both bio-based and biodegradable. Some fossil-derived plastics can also biodegrade and thus are also referred to as “bioplastics” [27]. Overall, plastics are classified into (1) bio-based and biodegradable; (2) bio-based and non-biodegradable; (3) fossil-derived biodegradable; and (4) conventional, fossil-derived, and non-biodegradable [29]. All of these plastics—sometimes referred to as “agri-plastics”—are used in agriculture and horticulture for crop and livestock production [30]. Mulch films, stretch films, silages, greenhouses, small tunnels, irrigation piles, drippers, non-woven nets, protective nets, and plant accessories (e.g., clips, supports, ties, etc.) are commonly used in crop production, whereas bale nets, twine, silages, and stretch films are used in livestock production [27,31]. Overall, the most frequently used plastics (typically single-use) are fossil-derived, non-biodegradable, and are manufactured from polymers such as low- and high-density polyethylene (LDPE, HDPE), polypropylene (PP), expanded polystyrene (EPS), ethylene-vinyl acetate copolymer (EVA), polyvinylchloride (PVC), and polyethylene terephthalate (PET) [30,32]. In the majority, conventional plastic mulch films are manufactured from low-density polyethylene (thin films) and propylene (thick films) [22,32,33,34]. According to Eunomia (2021), plastic mulch films are considered to be the second largest share of plastic films that are used in crop production in Europe [27]. After being used, plastic mulch films are difficult to dispose of, as prior to recycling, they need additional labor to be collected from the field and washed to remove the soil dirt. Recycling these films is often economically infeasible due to being heavily covered with soil and contaminated with pesticides and/or fertilizers. Similarly, most conventional plant accessories are likely to end up in the green waste stream. For example, single-use plant supports, clips, ties, and string are not separated from plant residues after harvesting and, together with the plant residues, are turned into composting facilities. This results in many difficulties for composting facilities and poses a threat of persistent microplastics in composts [22,27,30,35]. Therefore, the substitution of fossil-derived, non-biodegradable plastic mulch films and plant accessories with bio-based and biodegradable plastic alternatives, which would undergo biodegradation during industrial composting or in situ in soil, is fully justified [1,22,27,33]. In 2023, the global production capacity of bioplastics for agriculture and horticulture was 5% [36]. According to European Bioplastics (2024) [27], in view of the global bioplastics production capacities in 2022, 8% of certified soil-biodegradable materials were used for agricultural and horticultural applications. In 2027, it is expected that certified soil-biodegradable materials will reach 9% of global bioplastics production [27].

### 1.3. Bio-Based and Biodegradable Plastic Mulch Films and Plant Accessories

Due to the fact that conventional fossil-derived and non-biodegradable plastic mulch films and plant accessories (frequently single-use) are the most difficult to collect, separate from plant residues after harvesting, and then dispose of, it is justified to replace them with more sustainable bio-based and biodegradable alternatives. The most commonly used materials for the production of certified soil-biodegradable items for agriculture and horticulture are polyhydroxyalkanoates (PHAs), polybutylene adipate terephthalate (PBAT), polybutylene succinate (PBS), polylactic acid (PLA), polycaprolactone (PCL), and starch blends with increasing use of PHAs and PLA [20,32,36,37]. However, the production of bioplastics for crop production requires significant quantities of these biopolymers, which are still expensive. As a consequence, the market prices of bioplastic plant accessories or mulch films are higher than those of fossil-derived and non-biodegradable. One way to decrease the content of biopolymers and, at the same time, maintain or improve mechanical properties is to use additives and/or fillers. The fillers constitute a diverse and vast group of mineral and natural materials and typically perform two major functions when incorporated into composites/biocomposites, i.e., (1) reinforcing the properties of final products and/or (2) increasing the density and, thus, reducing the mass of virgin polymers/biopolymers in the final products [38]. Due to specific properties, biochar can also perform these functions and be used as a filler for developing plastics and bioplastics for agricultural and horticultural applications. Biochar can replace some of the commonly used fillers, in particular, inorganic fillers such as CaCO_3_, talc, glass fibers, or clay nanoparticles, as well as carbon-based fillers, e.g., carbon black, carbon nanotubes, or carbon fibers [19,39]. In the case of bioplastics, biochar used as a filler can offer additional functionalities to the final products, reduce the environmental impacts of plastics, and thus contribute to sustainability and circularity in horticulture [24].

### 1.4. Literature Screening

**Figure 1:**
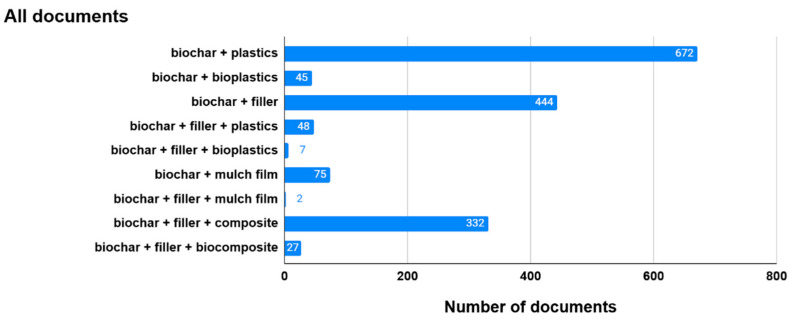
Literature screening of all publications on biochar narrowed down to the scope of this review (published until November 2024 and listed in WoS).

The literature screening performed through Web of Science of all types of documents on biochar using the keyword “biochar” yielded over 41,640 documents, including 15,306 documents with the word combination of “biochar” and “agriculture” and 824 with the word combination of “biochar” and “horticulture”. This demonstrates the overwhelming number of publications on biochar and its implications for agriculture and horticulture. The screening was narrowed down to the scope of this review, and the combinations of the following words were used: “biochar”, “plastics”, “bioplastic”, “filler”, “mulch film”, and “biocomposite” (Figure 1).

**Figure 2:**
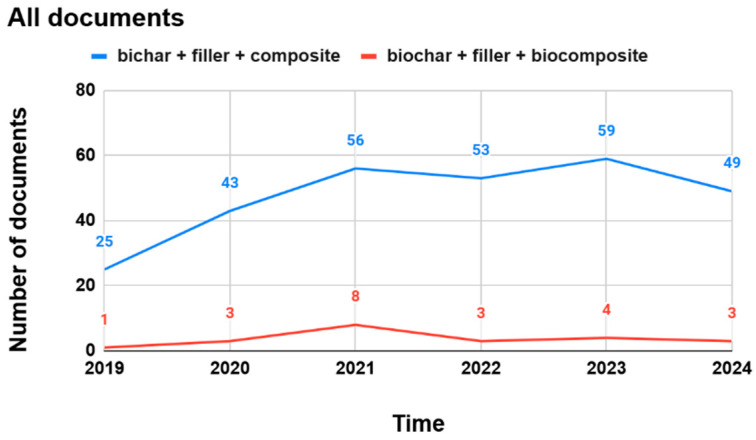
Literature screening of all publications on biochar used as a filler in composites or biocomposites during the last 5 years (published until November 2024 and listed in WoS).

Almost 82% of all documents with the word combination of “biochar” and “filler” were published within the last 5 years (after 2019). This indicates the increasing interest in biochar as a filler for producing plastics and/or bioplastics for various applications in a wide range of areas, including agriculture, horticulture, and other industries (e.g., automotive, construction, and building). We also looked into reviews with these word combinations. It turned out that 36 review papers with the word combination of “biochar” and “filler” were published, and only five review papers with the words “biochar”, “filler”, or “plastics”. No review papers were found with the word combination of “biochar”, “filler, “bioplastics”, “biochar”, “filler”, or “mulch film”. We also searched for word combinations of “biochar”, “filler”, “composite”, and “biocomposite”. The number of publications that addressed biochar as a filler in composites (i.e., conventional, fossil-derived) is significantly higher than the number of publications specifically focusing on biocomposites (i.e., renewable, bio-based). Using the word combination of “biochar”, “filler”, “and biocomposite”, the search yielded 27 articles during the period of the last 5 years (Figure 2).

The presented results obtained from the literature screening demonstrate that there has been a significant interest in biochar as a filler, specifically in composites (i.e., conventional, fossil-derived, non-biodegradable). But there is also some interest in biochar being incorporated into biocomposites (i.e., bio-based and biodegradable). These biochar-added composites and biocomposites can be used to manufacture various products for specific applications, including bioplastics for horticultural applications. Although biochar has applications in many areas, such as agriculture and horticulture, aerospace, automotive, packaging, and pharmaceutical industries [39], and this has already been demonstrated and reported in the literature, there is a significant gap to fill in on biochar as a renewable and bio-based filler for developing bio-based and biodegradable plastics to be used in horticulture. Despite the fact that literature reports some studies that tested the suitability of various types of biochars as fillers in plastics and bioplastics, mostly on a laboratory scale, there is very little information on the performance of biochar in prototypes or final products such as mulch films and plant accessories. Therefore, in this review, we look into the most recent knowledge on biochar as a filler for developing and manufacturing bioplastics, in particular for crop production, such as bio-based and biodegradable mulch films and plant accessories.

The overall goal of this review is to demonstrate the applications of biochar as a filler in bioplastics used in horticulture, summarize the most recent knowledge, and discuss future research directions. With the review of the current state of the art, we are addressing some of the most important issues, such as the requirements for biochar to be used as a filler for bio-based and biodegradable plastics, the effect of biochar properties and loading rates on mechanical properties, and suitability of biochar for manufacturing of bioplastics for horticultural applications. In addition, this review provides the current knowledge, identifies the gaps to be addressed in future research, discusses the advantages and limitations of biochar used as a filler in bioplastics, explains technological limitations and how to overcome them, and contains some recommendations for bioplastics manufacturers.

## 2. Requirements for a Filler to Be Used in Bioplastics

In general, fillers are materials of different origins and properties that are added to polymer/biopolymer composites, and they act as reinforcing or expanding agents, thus allowing the modification of the properties of plastics. Fillers constitute a vast group of materials that are of natural, mineral, metallic, or artificial origin. The effect of the filler addition in fossil-derived as well as bio-based composites on their properties depends on filler loading, particle size and distribution, distribution and dispersion, and shape and aspect ratio. The incorporation of fillers into plastics/bioplastics aims primarily to improve the mechanical strength of polymers/biopolymers and, thus, the mechanical properties of the final products. These properties will depend on many factors, including the type of polymer/biopolymer, the properties of the filler itself, and the loading rate [38].

Since most bio-based and biodegradable plastics for horticultural use are produced from biopolymers, the fillers incorporated into biopolymers to form biocomposites are often of natural origin. For example, flax fiber is often used in PLA-based composites. It can improve toughness at the loading of 30% [40]. PLA composites can also be filled in with kenaf fiber, which, if added in the amount of up to 70%, improves mechanical properties. Compared to other fillers, kenaf is usually added in significant quantities, reducing the consumption of a polymer [41]. Other fillers added to PLA include cellulose fibers, nano-cellulose, nano-cellulose fibers, and other fibers of natural origin. The purpose of their use is to improve mechanical properties and to reduce the amount of costly biopolymers [42,43,44,45]. The mechanical properties of PLA or other biodegradable polymers can be significantly improved by incorporating glass and carbon fibers as fillers. However, these fillers do not biodegrade and thus are not suitable for bioplastics designed to biodegrade in soil [42,46,47]. It has to be emphasized that bioplastics, specifically for horticultural uses, are rarely composed of a single polymer and are often based on polymer mixtures (e.g., PLA/PHA, PLA/PCL or PLA/PBS and PLA/PBAT) with suitable filler of natural or mineral origin [22,33,34,48].

Selection of a suitable filler to be used in bioplastics for horticultural applications should take into account the following criteria: (1) specific conditions for applications of bioplastics (e.g., type of soil, climate and weather conditions, temperature, type of crops, organic or conventional farming, etc.), (2) required properties of the final product (e.g., mechanical properties, design assumptions related to the geometry of the molding and wall thickness, durability, etc.), (3) requirements of manufacturing technology (e.g., additives and fillers used for biodegradable and bio-based polymers are sensitive to thermal loads caused by processing at elevated temperatures), and (4) end-of-life in open and controlled environments (e.g., in-soil biodegradability).

Despite the fact that there is a vast group of fillers that are already used in bioplastics for agricultural and horticultural uses, there is a growing interest in novel fillers that will not only allow the modification of bioplastics properties, reduce the costs of materials and manufacturing, but also will reduce the environmental impacts, contribute to zero pollution and assure sustainability and circularity of horticulture. Biochar is one of such novel fillers [19].

## 3. Biochar as a Filler in Bioplastics

**Figure 3:**
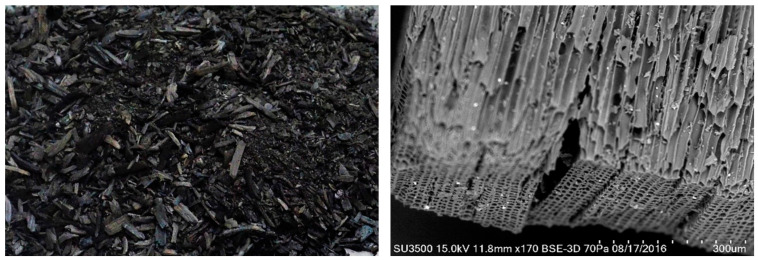
Wood-derived biochar.

Biochar is a porous, solid, carbon-rich material obtained through pyrolysis under temperatures of 300–700 °C in oxygen-depleted conditions of almost unlimited sources and types of feedstock materials (such as biomass and agricultural residues of plant and animal origin) [25,49,50] (Figure 3). The properties and applications of different biochars produced from a wide variety of feedstock under specific pyrolysis parameters (i.e., heating and residence time, temperature) have been extensively researched and reported in the literature. Types and characteristics of feedstock and pyrolysis parameters have a tremendous effect on the properties of biochar, including biochar yield and composition (in particular, carbon content), porosity, surface area, particle size, and the distribution and presence of functional groups (e.g., hydroxyl, carboxyl, and phenolic groups) [51,52,53].

Undoubtedly, the most common applications of biochar are observed in agriculture and horticulture [5,54]. Biochar can be directly applied to soil as an enhancer or indirectly as a fertilizing component or a microbial carrier to improve soil properties and fertility and increase crop yields [4,7,55]. Other applications of biochar include animal feeding and manure treatment through, e.g., composting or anaerobic digestion [2,3,56,57,58]. The recommendations for biochar to be sustainably produced and safely introduced into soils are provided by the European Biochar Certificate (EBC) [59]. Biochar is classified under the “Component Material Category” (CMC 14) by the EU Fertilising Product Regulation and thus can be used as a soil enhancer provided that it complies with the requirements and national regulations [60,61].

**Figure 4:**
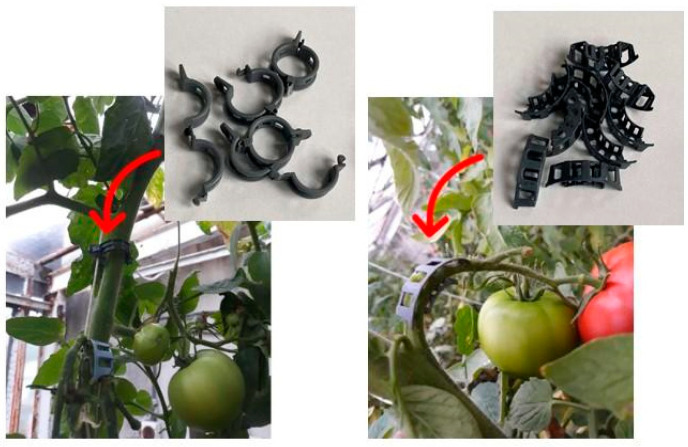
Plant accessories (plant supports and clips) with wood-derived biochar as a filler [22,33].

For the past decade, there has been a growing interest in biochar as a filler in plastics and bioplastics used in various areas, including agriculture and horticulture. First, studies on biochar as a potential additive/filler/reinforcement were initially performed for wood, polymer (polypropylene), malleated anhydride polypropylene composites [62], and wood polymer composites [63,64]. The idea of using biochar as an additive was primarily to reduce the quantities of organic waste and turn it into biochar and then to use biochar in wood/polymer composites to mitigate the effects of wood/plastics composites [63]. Other examples include the use of biochar from chopped miscanthus fiber, which was incorporated as a reinforcing filler into bio-based polyamide 6.10 to obtain composites for under-hood automotive parts [65]. In view of these, biochar has also been considered a promising filler to be incorporated into biocomposites for the production of bioplastics for horticultural uses. Figure 4 presents examples of plant accessories, such as plant supports and clips with wood-derived biochar as a filler, which was specifically developed for tomato cultivation [22].

As a filler in bioplastics for horticultural uses, biochar can (1) replace some biopolymers in biocomposites; be used as a (2) substitute, e.g., an inorganic filler; and (3) provide additional functionalities to enhance the performance of the final products when applied to soil. Examples of the application of biochar as a filler for the production of bioplastics for horticultural use have been reported in the literature. Table 1 presents examples of biochar used as a filler to produce typical bioplastics for agriculture and horticulture, such as mulch films, plant accessories, packaging, compost bags, etc.

In view of the reported studies, the addition of biochar to biopolymers generally resulted in a number of benefits, in particular, improved mechanical properties, stability, and flexibility. Also, biochar can replace traditional carbon black pigments in biodegradable composites and, when decomposed in soil, can act as a source of carbon. What is more, adding biochar to PLA and PHB improved the biodegradability of the biocomposites. Although there have been a number of reported benefits of using biochar as a filler in bioplastics, there are also disadvantages and limitations. It has to be pointed out that with the increasing loading of biochar in a biocomposite, the mechanical properties, such as tensile strength and elasticity, tend to deteriorate. According to the reported studies, biochar loading varies significantly and depends mostly on a biopolymer and its properties, the requirement for the final product and its applications, and the manufacturing method and parameters. Table 2 presents the biochar loading rates for selected biopolymers/mixtures of biopolymers for mulch films. For these bioplastics, different biochar loading rates of up to 20% were reported (by weight) [23,68]. In the case of mulch films, the loading of a filler affects the thickness of a film, which in turn influences the in-soil biodegradability of used mulch films.

According to Chang et al. (2021) [50], the following properties of biochar make it a promising filler for bioplastics: low density, porous structure and larger surface area, cation exchange capacity, functional groups, carbon structure, pH, and thermal and electrical conductivity. These biochar properties influence the manufacturing process and the performance of bioplastics in horticultural uses, e.g., in fields and greenhouses. For example, low-density biochar results in the reduced weight of biocomposites, porous structure, and relatively larger surface area and increases the interactions between a filler and a polymer/mixture of polymers, and the carbon content and structure increase mechanical properties of bioplastics [19,50]. Table 3 provides examples of some characteristics of biochars used for developing and manufacturing the most commonly used bioplastics, such as plant clips and supports and mulch films.

However, only a few studies reported in the literature provided sufficient information on biochar properties and how they impact the properties of biocomposites and the final products. Overall, biochar used as a filler in various bioplastics demonstrates a high content of carbon. Depending on the type of the final product, the particle size of biochar is in the range of 40–80 µm (for mulch films) and <1000–1500 (for various plant accessories).

## 4. Manufacturing of Biochar-Added Bioplastics

### 4.1. Incorporation of Biochar into Bioplastics

**Figure 5:**
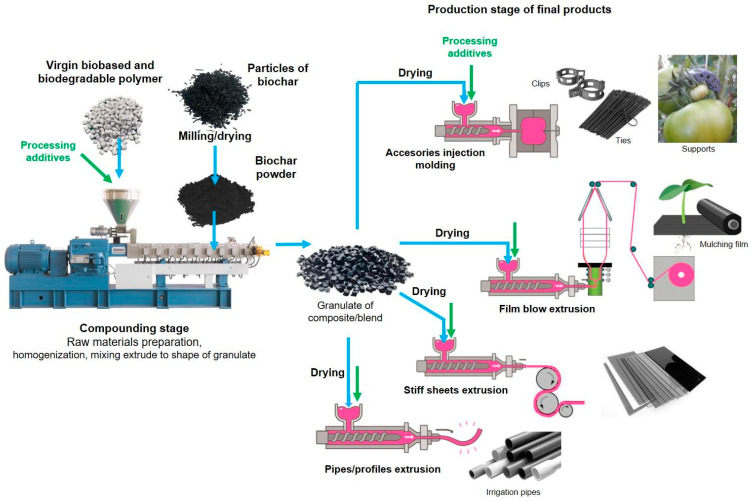
Schematic diagram for manufacturing of biochar-added bioplastics, including mulch films and plant accessories [22,33].

Manufacturing of biochar-added bioplastics is a complex process that generally covers two stages—(1) compounding and (2) processing (Figure 5)—and is performed by commonly used methods such as melt extrusion and injection molding.

Biochar that will be incorporated into bioplastics requires prior pretreatment, such as drying and milling. Due to the high risk of explosion, the process of grinding into powder should be carried out in special mills with temperature control. In the case of grinding biochar into dust, a liquid (e.g., water), which is a cooling medium and a carrier in the grinding processes, should be applied. It needs to be emphasized that the dimension of biochar particles is a very important factor connected with the rheological properties of the melted polymer. Lower biochar particle dimensions have an impact on the possibility of obtaining the lower thickness of the extruded film, so grinding is a crucial process of biochar preparation as a filler. Injection molding products that have a wall thickness higher than 1.5–2 mm can be produced with particles of biochar of 0.2–0.4 mm, but films with thicknesses of 0.040 mm need extremely small particles of biochar (less than 0.005 mm (5 microns)) [39]. Next, ground biochar in a fine form is used in the process of compounding. Prior to feeding ground biochar into the machine, biochar has to be dried. This also applies to all organic additives that absorb moisture from the environment. Biochar has special sorption properties, and due to its very large specific surface area, it can quickly absorb a significant amount of moisture from the air. Therefore, it is equally important to properly protect biochar after drying and before feeding it into the compounding machine. Due to the low stability of biochar at elevated temperatures, biochar should not be fed into the machine’s hopper, as is the case with the polymer matrix, but from side feeders into the part of the plasticizing system where the material is already plasticized. In the next part, only mutual mixing and homogenization of materials with each other takes place. The liquid mass is directed to the extrusion head and then granulated using a method depending on the viscosity of the obtained composite. Since cooling takes place in an aqueous environment, the obtained composite should be dried after the granulation process [33]. Before using the finished composite in the production processes of specific elements/products, it is necessary to ensure that the moisture content of the granulate does not exceed 0.15–0.2% by weight. Otherwise, the granulates should be subjected to a drying process, and it is recommended to use dry air dryers equipped with molecular sieves (i.e., air dehumidifiers) for this purpose. Production of individual products requires the selection of an appropriate technology that allows for the expected properties of the final products to be obtained. For this purpose, it is necessary to prepare and adjust, apart from the machine, appropriate equipment such as an injection mold, extrusion head, calenders, etc. [19,78,79].

The manufacturing of biochar-added bioplastics also requires the presence of various additives that are used to modify other physical and functional properties. These additives are usually added during the compounding process of granulates but also are added during the processing of these materials. Depending on the requirements of the final product, e.g., bio-based and biodegradable mulch film with specific use for organic horticulture, it must meet a number of standards, including those related to the migration of substances from the product to the environment, i.e., Specific Migration Limit (SML) [80]. For organic horticulture, it is required to ensure that the additives incorporated into, e.g., bio-based and biodegradable mulch films and other plastic components (e.g., haze agents, colorants, color batches, slip and anti-slip agents, and a number of other specific for a given group of products) do not pose any hazards to soil and crops during the process of in-soil biodegradation of these films. Therefore, tests are also performed for this group of products to determine specific and general migration and ecotoxicity of the soil in which the biodegradation of products takes place. None of the substances penetrating the soil can cause a negative impact.

### 4.2. Technological Challenges

Biochar as a filler is a very demanding material in terms of handling, storage, transportation, and processing. Primarily, this is due to its physical properties, such as moisture absorption, large specific surface, high surface hardness, and crushing hardness. Most of these properties make processing of biochar difficult from a technological perspective (e.g., wear of parts used in plasticizing systems as well as flow channels of injection molds). In view of this, it is not recommended to use hot-channel systems in feeding molding nests with liquid polymer [33]. The need to dry and maintain a very low level of moisture of biochar for processing, coupled with the simultaneous inconvenience caused by problematic drying and the risk of explosion as a mixture of dust with air, causes serious problems in biochar processing and preparation for processing. High dustiness also causes difficulties in the transport and cleaning of machines and all peripheral devices used in processing. EBC provides a set of guidelines for producers of biochar, the special focus of which is on safety and health issues [59]. However, there is insufficient information in the literature on scaling up the production of biochar-filled bioplastics and how to overcome the technological difficulties of using biochar as a filler during compounding and processing on the industrial scale. Therefore, there is a need to formulate guidelines for processors and manufacturers who use biochar as a filler in plastics/bioplastics to ensure the safe and proper use of technological infrastructure.

Other technological challenges are associated with the thermal stability of biopolymers. Most biomaterials demonstrate low thermal stability, which results in limited processing and requires careful selection of traditional processing machines and devices. Biomaterials do not need significant modifications during processing. However, there are two factors that may cause their thermal degradation: (1) processing temperature and (2) the time the material stays in the plasticizing systems. These are influenced by, among others, the length of the plasticizing unit and the applied plasticizing parameters. In the case of injection molding, individual values can be controlled independently; however, in the case of extrusion, increasing the screw rotation speed can generate greater shear stress and the generation of more heat, which, as a consequence, can contribute to the initiation of thermal degradation processes. A major challenge in biopolymer processing is to increase their thermal stability by reducing the impact of heat beyond that required for the plasticization and homogenization process. This could be achieved by (1) reducing the configuration of processing screws and lower L/D values than for thermoplastics (L/D = 18–20), (2) using a lower temperature profile of heaters section of the plasticization system (e.g., for PLA: temperatures not higher than 165–175 °C), (3) using effective (water) cooling of the plasticization system of extruders instead of air (fan), and (4) reducing shear stresses during plasticization processes by using lower rotational speeds. In addition, thermal stability can be improved in biopolymers by physical and chemical modification with additives that stabilize the processing (e.g., agents reducing internal friction) and by reducing the share of biopolymer by adding organic fillers (e.g., talc, chalk, plant-based fillers such as coconut fibers, flax fibers, sisal, and others). Prior to the use of any additive, its thermal stability should be determined, e.g., using the TGA or STA method. Some of the biodegradable polymers do not demonstrate stable thermal properties to start mass loss (TGA onset) in comparison to thermoplastic fossil-based polymers (e.g., PP, PE, PA) [81]. According to the literature, there has been a significant interest in improving the thermal and processing properties of biopolymers, in particular, by using physical and chemical modifications.

### 4.3. Human Health Risks

Although biochar has been extensively investigated, there are few reports on the safe use of biochar and its potential risk to human health. One of the biggest concerns about using biochar in the process of manufacturing biochar-added plastics on an industrial scale is related to long-term exposure to biochar-induced fine dust [82,83]. This dust can be formed during biochar production and transportation, as well as during preparation and incorporation into biocomposites. It has been reported that biochar (depending on the feedstock origin, composition, and pyrolysis parameters) can be a source of emissions of, e.g., volatile organic compounds [84]. According to the most recent studies, biochar-induced dust poses multiple health risks, mostly when very small particles are inhaled over a prolonged period of time. This can lead to respiratory diseases and even cancer [85]. Some studies pointed out that exposure to biochar particles with airborne pollutants can result in irritation of the eyes and mucus membranes [86]. Other potential risks are related to spontaneous ignition of biochar accumulations [87,88]. In their study, Dzonzi-Undi et al. (2012) [87] pointed out that biochar is susceptible to spontaneous ignition, and thus, there is a potential risk of explosion, in particular when biochar is stored, transported, or handled. Biochar self-ignition is associated with temperature and stockpile volumes. It was observed that at temperatures of 11 °C and below with high stockpile volumes, the risk of biochar self-heating does not occur. To prevent biochar from self-heating during the storage phase at various temperatures, it is recommended that the biochar void fraction be reduced, the stockpile volume be limited, and the initial temperature of biochar be decreased after the production process is completed [89]. According to the EBC standards on work safety and health, it is recommended—during transportation in bulk—to maintain the moisture content of biochar sufficient enough to prevent the generation and explosions of dust. It is mandatory to inform all workers about the potential risks and dangers related to biochar production [59].

## 5. Advantages and Limitations of Biochar as a Filler in Bioplastics

**Figure 6:**
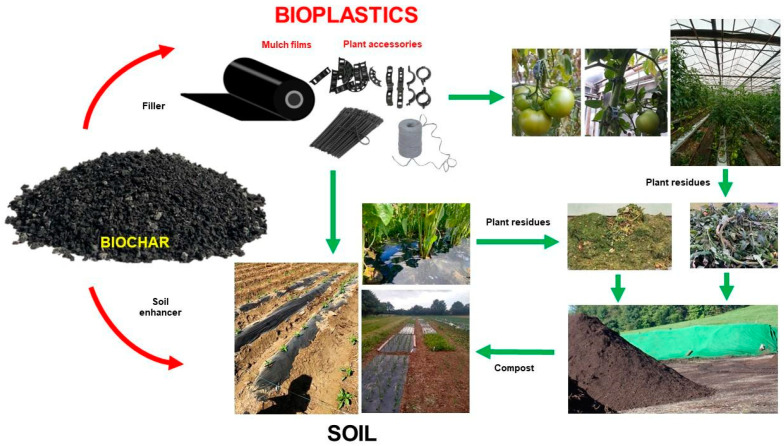
Biochar as a renewable and bio-based filler for bioplastics for horticultural uses [22,33,34].

Biochar offers substantial advantages as a filler in bio-based and biodegradable plant accessories such as mulch films, clips, and supports (Figure 6). Biochar-reinforced biocomposites demonstrate improved mechanical properties, including enhanced tensile strength, bending, thermal stability, flexibility, and electrical conductivity, compared to pure PLA (bio-based plastic). Additionally, biochar serves as a sustainable alternative to fossil fuel-based fillers, helping reduce the environmental impact of agricultural products by utilizing renewable materials that sequester carbon. Biochar also ensures processing stability during production and can be processed using conventional injection molding technology, making it suitable for large-scale manufacturing [21,22,68,69]. The reported studies (Table 1) highlight the key benefits of biochar, including its sustainability, compatibility with biodegradable matrices, potential as a soil enhancer, and cost-effectiveness compared to conventional fillers such as carbon black or carbon nanotubes. According to Malińska et al. (2022), biochar-added biocomposites are fully degradable in industrial composting conditions at 58 °C, leaving behind biochar, which acts as a soil amendment, enhancing soil health and providing potential fertilizing properties. Moreover, compost derived from used plant clips mixed with agricultural plant residues is safe for plants, with no observed phytotoxic effects [22]. Incorporating biochar into composites also offers functional advantages, such as delaying photo-oxidation when included in the PBAT matrix, which extends the functional life of agricultural accessories. Botta et al. (2021) demonstrated that biochar-enhanced composites are suitable for applications such as packaging films, including compostable bags. For instance, films with 5–10% biochar show an improved elastic modulus while retaining high strain values, making them ideal for agricultural packaging [69]. Biochar’s ability to reduce light transmittance in TPU films is another useful characteristic for agricultural applications that require light diffusion or protection. Furthermore, as reported by Mayakrishnan et al. (2022), PBS composites with biochar filling degrade completely within three weeks under enzymatic conditions, leaving behind biochar to contribute further to soil enhancement [70].

Despite these benefits, a number of challenges remain to be faced, such as variability in biochar properties depending on feedstock and processing methods, potential reductions in bioplastic thermal stability due to contaminants, and limited studies on the environmental implications of biochar across the lifecycle of bioplastics [19,39]. While biochar has shown potential in enhancing the properties of polymer composites, particularly bioplastics, ongoing research is necessary to optimize its incorporation to achieve consistent and desirable results. Key gaps remain in understanding the interactions between biochar and bioplastics, especially regarding their biodegradability and overall functionality in composite materials [19]. Recent studies emphasize the role of biochar in contributing to the end-of-life benefits of biodegradable polymers such as PHB (polyhydroxybutyrate) and PLA (polylactic acid). Biochar helps to produce carbon-neutral composite materials, lowering the environmental impact of plastics by acting as a sustainable filler while reinforcing their durability. These findings support the potential of biochar in circular economy models where materials are designed to minimize waste and ecological footprints [24]. Moreover, biochar has been shown to enhance the biodegradability of PLA and PHB composites, with studies confirming that the use of biochar as a filler up to 20% by weight results in complete enzymatic degradation, leaving behind biochar as a soil amendment [71].

The incorporation of biochar into biocomposites can lead to several changes in the material properties. When filled with 15% biochar, the tensile strength of the composite is reduced. This reduction can be attributed to the tendency of biochar to form agglomerates, which may negatively affect the mechanical properties of the resulting materials. As the biochar content increases, the extensibility of the composite decreases, indicating a loss in flexibility [21]. Additionally, the use of biochar in biocomposites may result in slower and incomplete decomposition when subjected to home composting, particularly when the composting temperatures are lower than in industry composting. This slower degradation process could lead to longer persistence of the material in the composting environment [22]. The addition of biochar also has an impact on the elasticity of the polymer matrix. Even a moderate increase in biochar content can reduce the material elasticity compared to the composite without biochar [23,69]. Specifically, the addition of 20% biochar significantly reduces the elasticity of a polymer. Furthermore, higher biochar loadings contribute to a decrease in the oxygen transmission rate of nanocomposite mulch films, which could affect the performance of these materials in agricultural applications [70]. Biochar in bioplastics for horticultural uses can enhance biodegradability and improve material properties. However, as biochar-filled materials degrade, there is a risk of producing microplastics, especially if the plastic matrix is not fully biodegradable. While biochar may accelerate degradation in some cases, incomplete breakdown under certain environmental conditions could still lead to microplastic formation, particularly in composting environments or when the material becomes brittle. Further research is needed to assess and mitigate the potential for microplastic generation from biochar-filled bioplastics [90]. More research is required to ensure that these materials fully degrade without contributing to microplastic pollution in soil and water [22,91]. Despite the advantages of using biochar as a filler, there are still critical areas requiring further investigation. These include the potential toxicity of biochar-based composites, their long-term environmental implications, and the variability of biochar properties due to differences in feedstock and processing conditions. Expanding research in these areas is crucial to fully leverage the ecological and functional benefits of biochar in bioplastic applications [24].

## 6. Summary and Outlook

In view of the current knowledge on the use of biochar as a filler in biocomposites for bio-based and biodegradable plastics, in particular for horticultural applications, biochar can be an alternative and a renewable filler in bioplastics such as mulch films or plant accessories. Biochar—depending on its origin, feedstock characteristics, and process parameters of pyrolysis—has demonstrated the potential as a filler for developing fossil-derived as well as bio-based and biodegradable plastics. It can be used for reinforcement as well as the reduction of the weight of virgin polymers/biopolymers. What is more, biochar can provide additional functionalities (e.g., a carrier for fertilizers, a black colorant) to bioplastics and thus make them more suitable for horticultural applications, taking into account the end-of-life (i.e., in-soil biodegradability). According to the reported studies, the following properties of biochar, i.e., carbon content and structure, porosity and surface area, and low density, are considered important for manufacturing bioplastics. These biochar properties and loading rates influence the final properties of bioplastics, such as the weight of composites and the mechanical properties of final products. Biochar loading depends on the type of a product and its properties. Generally, with the increase in biochar loading, the mechanical properties deteriorate. Incorporating biochar through compounding into biocomposites (granulates) for further processing and manufacturing (e.g., injection molding, blow extrusion) of bioplastics such as mulch films or plant accessories poses many technological challenges related to pretreating biochar (i.e., drying, milling, mixing, homogenizing) and processing and manufacturing final items (i.e., feeding the biocomposite into injection molding or extrusion systems). Moreover, processing biochar-added composites in technological units leads to contamination of, e.g., flow channels, which need to be cleaned. It has to be emphasized that biochar forms fine dust during handling, storing, transporting, and processing and, thus, can pose potential risks to human health through inhalation and the danger of, e.g., self-heating and explosion.

Despite the fact that there are a number of reported studies investigating the use of biochar as a filler for plastics and/or bioplastics, there is insufficient information on the properties of biochar incorporated into bioplastics and the effect of biochar loading rates on properties and field performance of the final products, i.e., prototypes of mulch films or plant accessories. In the literature, biochar is presented as a “green’, “sustainable”, “circular”, and “renewable” filler. The reasons for that are that biochar is obtained from waste biomass and agricultural residues, demonstrates promising properties as a filler for bio-based and in-soil biodegradable plastics, and can substitute commonly used mineral fillers such as carbon black and thus contribute to sustainability and circularity in crop production. However, further research on the analysis of environmental impacts, sustainability, and circularity is needed to support these claims.

The use of biochar as a new, renewable, bio-based filler, specifically for manufacturing bioplastics for horticultural uses, such as mulch films and plant accessories, will be driven by changes in the regulations. Biochar has already been included in the Fertilising Products Regulation (FPR) 2019/1009 under the “Component Material Category (CMC) 14”, and, as such, can function as a soil enhancer. However, it has to be emphasized that biochar for soil applications should fulfill the quality and safety requirements. Just recently, soil biodegradable plastic mulch films were incorporated into the EU Fertilising Products Regulation (FPR) 2019/1009 under the “Component Material Category (CMC) 9”, “other polymers”, and included in the “Product Function Category (PFC)” as a soil improver (PFC 3), in particular as an inorganic soil improver (PFC 3B) [27,28]. According to the FPR, a soil improver is a fertilizing product that “*means a substance, mixture, microorganism or any other material, applied or intended to be applied on plants or their rhizosphere or on mushrooms or their mycosphere, or intended to constitute the rhizosphere or mycosphere, either on its own or mixed with another material, for the purpose of providing the plants or mushrooms with nutrient or improving their nutrition efficiency*”, and its function is to “*maintain, improve or protect the physical or chemical properties, the structure or the biological activity of the soil to which it is added*” [92]. Since *“mulch films are used to maintain, improve or protect the physical or chemical properties, the structure or the biological activity of the soil”,* with this inclusion, they comply with the definition of a soil improver [28]. This means that soil biodegradable plastic mulch films—provided that they are in compliance with the biodegradability criteria set in the Delegated Regulation 2024/2787 of 23 July 2024—are now classified as soil improvers and can be treated as safe for the environment and contribute to soil health and soil fertility [27,93,94]. With this inclusion, it is expected that the market of in-soil biodegradable mulch films will develop. In view of this, it is anticipated that the interest in biochar as a renewable and bio-based filler for bioplastics for horticultural applications will also increase.

## Figures and Tables

**Table 1:** Examples of biochar use in bioplastics for horticultural use.

Biochar	Composite	Bio-Based/ Fossil Polymers	Improved Properties	Obtained Products	Limitations	Ref.
Wood residue-derived biochar	PBAT (EcoWorld^®^)	bio-based	An alternative solution to the use of fossil fuel-based fillers.	Mulch film	Reduced tensile strength when filled with 15% biochar.	[66]
Wood residue-derived biochar at 550 °C	Polylactic acid (PLA Ingeo Biopolymer 4043D manufactured NatureWorks LLC) and BIOPLAST GS2189 (BIO)	bio-based	Biochar-reinforced biocomposites exhibited stability during processing and can be produced using conventional injection molding technology for thermoplastic polymers.	Plant clips and supports	Biochars tend to form agglomerates, which may have a negative impact on the mechanical properties of various accessories manufactured from these biocomposites.	[21]
Sewage sludge-derived biochar at 550 °C						
Bamboo-derived biochar (commercial option)	PLA	bio-based	Composites with added biochar showed increased tensile, bending, and flexibility properties compared to pure PLA.	Agriculture accessories	The greater the biochar filling, the lower the extensibility of the composite.	[67]
Wood-derived biochar at 550 °C	Bioplast GS2189	bio-based	Fully degradable in industrial composting (at 58 °C). Compost obtained by mixing used clips and supports with agricultural residues is safe in terms of phytotoxicity for plants.	Plant clips and supports	Slower and incomplete decomposition of used clips and supports in home composting where the temperature reached only 30 °C.	[22]
Agricultural carob waste-derived biochar at 280, 340, and 400 °C	PBAT, commercial Ecoflex^®^ F Blend C1200, BASF, SE, Ludwigshafen, Germany	bio-based	Biochar particles in composites can benefit from the photo-oxidation delay of the PBAT matrix.	Packaging and mulch films	Reduced tensile strength (to some extent compared to the composite itself).	[23]
Wood biomass-derived biochar (commercial option)	PBSA pellets (BioPBSTM FD92PM)	bio-based	Biochar can replace traditional carbon black pigments in biodegradable PBSA composites with potential fertilizing properties for agriculture.	Mulch films, bag liners, and plant pots	The addition of biochar reduces the elasticity of the polymer (to some extent, compared to the composite itself).	[68]
Birch and beech wood-derived biochar (commercial option)	PBAT	bio-based	Blown films exhibited mechanical properties suitable for potential use as packaging film, in agriculture, and in compostable bags. The addition of 5 and 10% of biochar improved the elastic modulus while maintaining high strain values.	Agricultural and compost bag applications	The addition of 20% biochar reduces the elasticity of the polymer.	[69]
Digitalis purpurea-derived biochar at 400 °C	TPU	bio-based	The light transmittance value of the TPU film alone was 82%. After adding 2% biochar filling, the transmittance value dropped significantly to 65%.	Mulch films	Increased biochar filling load decreased the oxygen transmission rate of nanocomposite mulch films.	[70]
Wood-derived biochar (commercial option)	Polybutylene succinate (PBS)	bio-based	The PBS composite with biochar filling was completely enzymatically degraded within 3 weeks, and the degradation product, i.e., the biochar itself, remained in the soil, providing a soil amendment.	Mulch films	Not found.	[71]
Miscanthus straw-derived biochar at 700 °C	Poly(butylene succinate) (PBS)	bio-based	The higher the biochar content, the faster the composite degrades.	Mulch film	Not found.	[72]
Wood-derived biochar	HDPE	fossil polymers	Better carbon sequestration in HDPE and rHDPE production.	Agriculture/ horticulture use	More research is needed; large amounts of 40–50% biochar are required to reduce the release of carbon dioxide during the production of fossil polymers.	[24]
	rHDPE					
	PLA	bio-based	10–20% biochar addition improves the biodegradability of PLA and PHB.			
	PHB					
Wood (stems of *Caragana korshinskii*)-derived biochar at 600 °C	polytetrafluoroethylene (PTFE)	fossil polymers	Biochar composite films were more degradable than PE films.	Mulch films	The 6% and 8% biochar content caused uneven stress distribution and deteriorating mechanical properties of the film compared to the 2% biochar filler.	[73]

**Table 2:** Examples of biochar-added mulch films investigated and reported in the literature.

Bioplastics	Biochar Type	Biochar Loading [wt %]	Biopolymer	Fabrication	Lab Scale Testing	Prototype Testing	Ref.
Mulch film (thickness 200 μm)	Agricultural carob waste-derived biochar	10, 20	PBAT, commercial Ecoflex^®^ F Blend C1200, BASF, SE, Ludwigshafen, Germany (bio-based)	Melt mixing and compression pressure of 1500 psi for 5 min at 170 °C molding.	yes	yes	[23]
Mulch film (planned analyses)	Wood biomass-derived biochar (commercial option)	5, 10, 15, 20	PBSA pellets (BioPBSTM FD92PM) (bio-based)	Single-screw rotated at a constant 60 rpm (23 Nm of torque).	yes	no	[68]
Mulch film (thickness 40 μm)	*Digitalis purpurea*-derived biochar	2, 4, 6, 8, 10	TPU (bio-based)	TPU and biochar nanocomposites were extruded, cooled, granulated, and dried at a head temperature of 180 °C and a screw speed of 110 rpm.	yes	no	[70]
Mulch film (thickness 80 nm)	Miscanthus straw-derived biochar	1, 2, 5	PBS (bio-based)	In situ polymerization method with 1, 2.5, and 5 wt % biochar.	yes	no	[72]
Mulch film (thickness, no data)	Birch and beech wood-derived biochar	5, 10	PBAT (bio-based)	Mulch film was obtained by compression molding.	yes	no	[69]
Mulch film (thickness 25–30 μm)	Wood residue-derived biochar	12–15	PBAT (bio-based)	Mulch film was extruded.	yes	yes	[66]
Mulch film (thickness 20 μm)	Bamboo wood-derived biochar	5, 10	PLA (bio-based)	Biocomposites were extruded using a twin-screw extruder.	yes	yes	[67]
Mulch film (thickness 20–25 μm)	Wood-derived biochar	5, 10	PBS (bio-based)	Extrusion of PBS/BC composite at 100 °C.	yes	yes	[71]
Mulch film (thickness, no data)	Wood (stems of *Caragana korshinskii)*-derived biochar	2, 4, 6, 8	PTFE (fossil polymer)	The biocomposite with biochar was prepared by dissolving gutta-percha, adding biochar, mixing, casting the mixture into a mold, and drying at room temperature.	yes	no	[73,74]
Mulch film (thickness 204, 209, 214 μm)	Raw cow manure-derived biochar	0.5, 1, 5	PVP (fossil polymer)	The films were prepared using the casting method. A PVP solution was first dissolved in water at 80 °C, followed by the addition of glycerol. Separately, arabic gum was dissolved in water, and synthetic zeolite, along with varying amounts of processed biochar, was added to this solution. The contents of both mixtures were then combined, homogenized, and poured into silicone molds.	yes	yes	[75]

**Table 3:** Examples of characteristics of biochars used as fillers in bio-based and biodegradable plant accessories and mulch films.

Bioplastics	Biochar Feedstock	Particle Size [µm]	Pyrolysis Temperature [°C]	Surface Area [m^2^/g]	Total Carbon Content [%]	Filler Content in Bioplastics [wt %]	Ref.
Clips and supports	Wood residue-derived biochar	<1500	550	7.2	81.4	10, 20	[21,76]
	Sewage sludge-derived biochar	<1500	550	335.9	42.2	10, 20	
Clips and supports	Wood derived-biochar	<1500	550	7.2	81.4	10, 20	[22]
Mulch film	Agricultural carob waste-derived biochar	13–60	280, 340, 400	-	65–73	10, 20	[23,77]
Mulch film	Wood biomass-derived biochar (commercial option)	5–60	-	-	67–75	5, 10, 15, 20	[68]
Agriculture accessories	Birch and beech wood-derived biochar (commercial option	<1000	-	-	-	5, 10	[69]
Mulch film	*Digitalis purpurea*-derived biochar	80	400	-	-	2, 4, 6, 8, 10	[70]
Mulch film	Miscanthus straw-derived biochar	<1000	700	-	-	1, 2, 5	[72]
Mulch film	Wood (stems of *Caragana korshinskii)*-derived biochar	40–60	300, 400, 500, 600	0.56, 2.41, 181, 381.5	68, 81, 86, 91	2, 4, 6, 8	[73,74]
Agriculture accessories	Wood waste-derived biochar	<1000	400–450	1.2, 1.6	68, 71	6, 24	[62]
Mulch film	Raw cow manure-derived biochar	1–50	-	-	48	0.5, 1, 5	[75]
Agriculture accessories	Wood-derived biochar	10	-	-	85	20, 40	[24]

## Data Availability

No new data were generated for this review paper. Bibliographic summaries were generated using search engines in Web of Science.
